# Caregiver Perceptions of Blood Glucose Monitoring in Children and Adolescents Living With Type 1 Diabetes in Kyrgyzstan, Mali, Tanzania, and Peru: Qualitative Study

**DOI:** 10.2196/98957

**Published:** 2026-11-09

**Authors:** Sofia Pfeffer, Elvis Safary, Aida Abraimova, Stéphane Besançon, Asel Dunganova, Cathy Haldane, Maria Lazo-Porras, Molly Lepeska, Moise Nguemeni, Silvana Perez-Leon, Kaushik Ramaiya, Priyanka Singh, Beatrice Vetter, David Beran

**Affiliations:** 1 University of Geneva, Rue Gabrielle-Perret-Gentil 6, Geneva, Geneva, 1211, Switzerland, 41 223729503, 41 223729504; 2 FIND, Geneva, Geneva, Switzerland; 3 Health Policy Analysis Center, Bishkek, Bishkek, Kyrgyzstan; 4 Santé Diabète, Bamako, Mali and Laboratoire MESuRS (Modélisation, épidémiologie et surveillance des risques sanitaires) au Conservatoire National des Arts et Métiers (CNAM), Paris, France; 5 Universidad Peruana Cayetano Heredia, Lima, Lima Province, Peru; 6 Health Action International, Amsterdam, North Holland, The Netherlands; 7 Santé Diabète, Bamako, Bamako, Mali; 8 Shree Hindu Mandal Hospital, Dar es Salaam, Dar es Salaam, United Republic of Tanzania

**Keywords:** type 1 diabetes, blood glucose monitoring, access, co-design, person centered

## Abstract

**Background:**

Caregivers play an important role in caring for children and adolescents with type 1 diabetes. Controlling blood glucose levels is an essential part of diabetes management. Previous studies in low- and middle-income countries have found that tools for measuring blood glucose are unavailable and unaffordable. However, very little is known about the perceptions of caregivers in low- and middle-income countries with regard to the important element of self-monitoring of blood glucose in type 1 diabetes and emerging technologies and their suitability for these contexts.

**Objective:**

The aim of this study was to explore caregivers’ perceptions of how self-monitoring of blood glucose impacts everyday life. For the purpose of this study, the term “caregiver” refers to either a parent or an adult responsible for assisting with the management of type 1 diabetes in a child/adolescent.

**Methods:**

This qualitative descriptive study involved 24 interviews with caregivers identified through a mix of purposive and snowball sampling. Interviews were carried out based on common themes from an interview guide. This study was part of a larger study conducted in Kyrgyzstan, Mali, Peru, and Tanzania. Interviews were analyzed using thematic analysis.

**Results:**

A total of 24 interviews were conducted, with over 80% of caregivers being female. The tools used raised issues related to blood glucose meters, such as finger pricks, follow-up, and affordability. Whereas for continuous glucose monitors, issues of acceptability, accessibility, and affordability of these devices were mentioned by the interviewees. Interviewees also highlighted different disruptions to daily life due to type 1 diabetes, such as nutrition, school, and the need to access medical care. Across settings, caregivers described substantial psychological burden linked to repeated finger pricks, financial strain, and challenges managing diabetes during school and nighttime periods.

**Conclusions:**

Caregivers’ perspectives on blood glucose monitoring highlighted the psychological and financial burden that weighs on them. Technological solutions, such as continuous glucose monitors, make it possible to alleviate certain obstacles related to the use of blood glucose meters and strips, but given their high cost, this can increase the financial burden.

## Introduction

In 2017, there were an estimated 9 million people living with type 1 diabetes, with 27% in low- and middle-income countries (LMICs) [1]. Diabetes management focuses on maintaining blood glucose levels within a safe range to avoid acute hypoglycemia related to insulin therapy, as well as long-term microvascular and macrovascular complications correlated to the pathophysiology of chronic hyperglycemia [2,3]. Diabetes complications represent important determinants of lower quality of life, demands on the health system, economic costs, and increased morbidity and mortality [4,5]. To manage blood glucose within daily insulin regimens, self-monitoring of blood glucose (SMBG) is necessary [6,7]. Different tools, such as blood glucose meters (BGMs) and continuous glucose monitors (CGMs), help people with type 1 diabetes manage their insulin doses and the body’s reaction to food, physical activity, and glucose patterns and trends. BGMs measure capillary blood glucose using a small drop of blood obtained by finger prick and applied to a test strip, giving the person their blood glucose level at a specific point in time [8]. Guidance proposes up to 8 tests per day at different times, such as before and after meals, at bedtime, around exercise, or when the individual suspects high or low blood sugar levels requiring them to act accordingly [6,7]. CGMs use a sensor that measures glucose in the interstitial fluid at frequent intervals, providing near-continuous information on glucose levels, direction and rate of change, and patterns over time [8]. These systems can also include alerts and transmit readings to smartphones for the individual or their caregivers.

Beyond measuring blood glucose, BGMs and CGMs have also been described as providing a sense of security and confidence, which is beneficial for mental well-being [9]. These tools also serve as a crucial tool for coordinating and sharing data with caregivers and health care professionals to enable effective treatment modifications and education [6,7,10]. This requires both the availability and affordability of these tools, as well as access to education and support from the health system, which in many LMICs remains problematic [10-12]. A market report carried out in LMICs showed that there are still a number of factors that limit access to blood glucose monitoring devices, such as product characteristics, quality standards, cost and pricing, financing for product procurement, distribution and supply chain, and provision of care [13].

Children and adolescents represent a specific population at risk of developing diabetes complications due to the complexity of diabetes management during this period [14]. Current recommendations from the International Society for Pediatric and Adolescent Diabetes indicate that glycated hemoglobin targets for youth living with diabetes should be below 7% [6]. The International Society for Pediatric and Adolescent Diabetes also recommends a target of 4 to 10 mmol/L for blood glucose levels over time within the target range of CGMs [15]. For children and adolescents, parents or caregivers play an important role in care [16]. According to Markowitz et al [16], the allocation of different diabetes-related tasks goes through an evolution as the child develops through early childhood, middle childhood/school-age years, and adolescence. In parallel to the changing roles, the relationship between the parent/caregiver and child also changes with the needs, maturity, and empowerment of the child and adolescent [14]. These factors all place a certain level of stress on the parent/caregiver. It has been shown that psychosocial factors such as depression and family conflicts are common among young people with diabetes and can lead to suboptimal management of their diabetes [17,18]. CGMs have been reported as playing a particularly valuable role in monitoring blood glucose in children and adolescents, providing notable benefits for the psychosocial well-being of young patients as well as their families [15]. However, these technologies are often unavailable and unaffordable in LMICs [19].

Previous studies have examined the availability and affordability of glucose monitoring devices and health system constraints in LMICs [11,12,19,20]; however, very little is known about the perceptions of caregivers in LMICs with regard to the important element of SMBG and emerging technologies such as CGM and their suitability for these contexts. Therefore, the aim of this paper is to explore caregivers’ perceptions on how SMBG impacts everyday life. For the purpose of this study, the term caregiver is used referring to either a parent or an adult responsible for assisting with the management of type 1 diabetes in a child/adolescent.

## Methods

### Study Design and Context

This qualitative study is part of a larger study [21], which aimed to define requirements for noninvasive and minimally invasive glucose monitoring devices in LMICs to inform a target product profile to guide the development and selection of suitable devices. Sampling, recruitment, training, and data collection were carried out in Kyrgyzstan, Mali, Peru, and Tanzania by research teams. This study is an analysis of caregiver perceptions from a series of interviews included within the wider study.

### Setting and Participants

Participants were selected through a mix of purposive and snowball sampling through diabetes associations and other diabetes-related networks in Bishkek (Kyrgyzstan), Bamako (Mali), Lima (Peru), and Dar es Salaam (Tanzania). Table 1 provides the specifics of each country. This study focused on 6 interviews per country.

**Table 1.** Description of each country.

Characteristic	Kyrgyzstan	Mali	Peru	Tanzania
Geographical location	Central Asia	West Africa	South America	East Africa
Population, total (2023) [22]	7,100,800	23,293,698	34,352,719	67,438,106
Life expectancy at birth (total years; 2022) [22]	72	59	73	67
Poverty headcount ratio at US $2.15 a day (% of population; 2017) [22]	0.3	20.8	2.7	44.9
Diabetes-related health expenditure per person, US $ (2024) [23]	1290	141	1318	153
Type 1 diabetes across all age groups, n [23]	2557	1424	19,247	24,955
Regions included in assessment	Bishkek (capital)	Bamako (capital)	Lima (capital)	Dar es Salaam (main urban area)

### Data Collection

This qualitative study collected data from interviews with a caregiver of a minor living with type 1 diabetes. Interviews were carried out by local teams with experience in qualitative research who were trained in person or online by a consultant hired by the project. Caregivers had to be the legal guardians of the minor. The interview guide consisted of a range of open-ended questions grouped into themes in order to respond to the objectives raised by the main study [21]. Some basic background information was collected from each interviewee, including gender of interviewee, gender of minor with type 1 diabetes, and self-reported income level. In the interviews, the following themes were discussed: diabetes, control of diabetes, the role of technology, glucose monitoring devices, requirements, societal, clinical, and technological factors, and cost. For some interviews, visual aids were used to help informants with insufficient knowledge of noninvasive and minimally invasive glucose monitoring devices to understand the type of technologies of interest in this study. The interviews were conducted in the local language, audio recorded, and transcribed. Interviews from Kyrgyzstan (in Russian) and Tanzania (in Swahili) were translated into English. The interviews from Mali (in French) and Peru (in Spanish) were not translated, but quotes were translated into English for the purpose of this publication by SP, who is bilingual in French and Spanish, and checked by DB (whose mother tongue is French and who is fluent in Spanish).

### Analysis

All interviews were reviewed by the lead author using reflexive thematic analysis, guided by Braun and Clarke’s 6-phase framework [24] as well as further reflections on this approach proposed by the same authors [25,26].

First, the lead author became familiar with the dataset through repeated reading of all transcripts with coding of relevant information linked to the research question. The coding combined a deductive approach using different domains included in the interview guide, such as blood glucose monitoring practices, device characteristics, social and clinical considerations, and cost, combined with inductive identification of patterns arising from participants’ accounts. Coding was primarily semantic, focusing on the explicit meanings conveyed by participants. On the basis of these codes, the data were grouped into recurring themes across interviewees and countries to organize the information. This was done manually using Microsoft Excel. Once an initial analysis was prepared, the interviews were reread and discussed between the lead author and DB. This process also enabled quotes to be retained according to their relevance and contributions based on the themes that emerged. Quotes were coded with a letter and a number to identify the respondent. The letter corresponds to the first letter of the country where the interview was carried out, and the number defines the different interviews. The characteristics of study participants were grouped, summarized, and presented descriptively. Involvement of colleagues from each of the countries who were involved in the data collection and overall study helped in both reviewing and defining potential themes. To detail the process of reporting the results, Table 2 details these using the guidance proposed by Braun and Clarke [26].

**Table 2.** Reporting on the analytical process.

Component proposed by Braun and Clarke [26]	Study approach
Type of thematic analysis	Reflexive thematic analysis was done through the exploration of caregivers’ knowledge and experience of blood glucose monitoring.
Theoretical orientation	Interpretive orientation was done with the research team interpreting the experience of the interviewees in their given context
Analytic focus	The analysis focused on caregivers’ reported experiences and perceptions.
Inductive or deductive orientation	Coding combined deductive attention to the study objectives and themes within the interview guide with inductive identification of topics not initially conceptualized.
Semantic or latent coding	Semantic coding focused on the accounts given by the interviewees.
Researcher familiarization	Lead author read each transcript several times and recorded preliminary analytic observations. DB reviewed this. Each country team was also familiar with their specific dataset and provided comments and input.
Coding process	Relevant segments were coded manually across the complete dataset using Microsoft Excel.
Researcher involvement	The lead author conducted the initial coding and developed candidate themes in discussion with the senior author. All other authors were involved in the design, implementation, and final analysis of the data.
Development of themes	Three overarching themes were identified: BGMs^a^, CGMs^b^, and impact on everyday life. Two themes related directly to the actual technology, whereas the third theme explored the impact of blood glucose monitoring regardless of the technology.
Review and refinement	An iterative process was taken reviewing themes against the transcripts as well as discussions between the lead and senior authors.
Definition of themes	This was done by reviewing the codes and looking at the interlinkages between these.
Writing and quotation selection	Illustrative quotations were selected to demonstrate the central meaning of each theme and relevant variation across settings.
Reflexivity	The main analysis was conducted by a junior researcher who, during their medical training, had worked on the topic of diabetes in LMICs^c^. This was complemented by the senior author, who has vast experience in the area. This allowed for, on the one hand, a “fresh” view of the issue to be confronted by one that has been shaped by more than 20 years of research. These two perspectives were complemented by the wide range of experience brought by the other coauthors.
Collaboration	This collaboration had multiple levels between international and national experts, as well as between junior and senior researchers, in the preparation of this paper.
Quality coherence	This was done through the coding and theme development process and discussions between the lead and senior author, combined with inputs from other colleagues and referring back to the dataset throughout the process.

a BGM: blood glucose meter.

b CGM: continuous glucose monitor.

c LMIC: low- and middle-income country.

### Ethical Considerations

The study protocol [21] was approved by ethics review boards in each country. The respective ethics committees and reference numbers are listed below:

- Kyrgyzstan: Ethics Committee at the Research and Production Association “Preventive Medicine” of the Kyrgyz Republic Ministry of Health; extract from the protocol number 9
- Mali: Comité d’Ethique de l’Université des Sciences, des Techniques et des Technologies de Bamako; number 2022/36/CE/USTTB
- Peru: Comité Institucional de Ética en Investigación de la Universidad Peruana Cayetano Heredia; 207529
- Tanzania: Muhimbili University of Health and Allied Sciences Research and Ethics Committee; MUHAS-REC-05-2022-1153

The overall study was sponsored by FIND (Foundation for Innovative Diagnostics, Geneva, Switzerland) and Health Action International (Amsterdam, Netherlands) as part of the ACCISS (Addressing the Challenge and Constraints of Insulin Sources and Supply) Study. The interviews took place after obtaining informed consent from the caregiver or legal guardian of the minor.

## Results

### Caregiver and Child Characteristics

Overall, 83.3% (20/24) of caregivers were women and 16.7% (4/24) were men. The children and adolescents were aged between 4 and 18 years old. Twenty-one interviews were done with a single caregiver and their respective child. The other 2 included a retired woman who looks after 11 minors with diabetes aged between 5 and 22 years old (T2) and T6, who cared for 2 children living with type 1 diabetes. Table 3 details some of the characteristics of the caregiver and child or children they cared for.

Self-reported income status revealed that none of the caregivers felt they belonged to a high-income household, 4% (1/24) to a middle-high*–*income household (P2), 29% (7/24) to a middle-income household (K1-5, M3, P1), 25% (6/24) to a middle-low*–*income household (K6, T2, T3, M5, M6, P5), 33% (8/24) to a low-income household (T1, T4, T5, T6, M4, P3, P4, P6), and 8% (2/24) to a very low*–*income household (M1, M2).

Among the methods used to measure blood glucose levels, it was found that the interviewees in Mali and Tanzania all used BGMs, while in Kyrgyzstan everyone used CGMs. In Peru, some used BGMs (P3, P4, P5, and P6) and others used CGMs (P1 and P2).

The results of the perceptions reported by interviewees will be presented according to the themes and subthemes that emerged in relation to blood glucose monitoring, which are illustrated in Figure 1. The overall impact of blood glucose monitoring on everyday life is presented, followed by the impacts of BGMs and CGMs in addressing some of these challenges.

**Table 3.** Characteristics of respondents.

Country and caregiver sex	Caregiver relationship to child	Child/adolescent age (y)	Monitoring method
Kyrgyzstan			
Female	Mother	12	CGM^a^
Female	Mother	7	CGM
Female	Mother	9	CGM
Female	Mother	8	CGM
Female	Mother	4	CGM
Female	Mother	12	CGM
Mali			
Female	Mother	16	BGM^b^
Female	Mother	17	BGM
Male	Father	17	BGM
Male	Father	15	BGM
Female	Mother	16	BGM
Female	Mother	15	BGM
Peru			
Female	Mother	16	BGM
Female	Mother	11	CGM
Female	Mother	12	BGM
Female	Mother	12	BGM
Female	Mother	9	CGM
Female	Mother	8	BGM
Tanzania			
Male	Father	No age mentioned, but caregiver has 2 children with type 1 diabetes	BGM
Female	Mother	13	BGM
Female	Mother	8	BGM
Male	Father	18	BGM
Female	Guardian	Woman cares for 11 minors with diabetes aged between 5 and 22 years	BGM
Female	Mother	15	BGM

a CGM: continuous glucose monitor.

b BGM: blood glucose meter.

**Figure 1.**
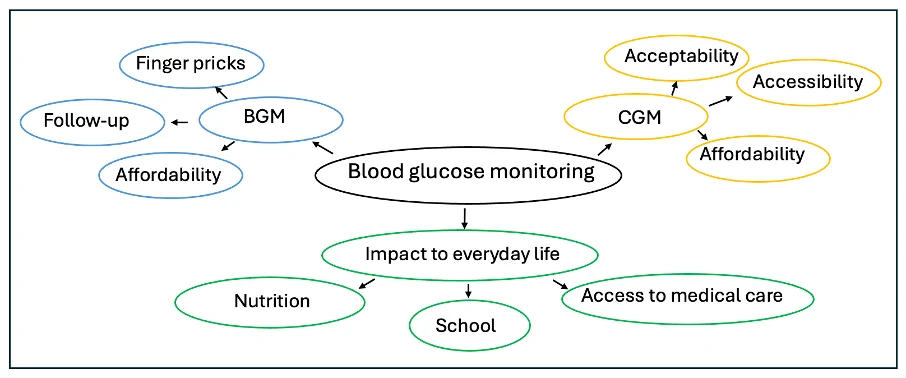
Themes emerging from the interviews. BGM: blood glucose meter; CGM: continuous glucose monitor.

### BGMs

SMBG using a BGM device was the main way of monitoring blood glucose, with 58% (14/24) of all interviewees using this method. The interviews highlight different challenges with regard to the use of BGMs, including the issue of finger pricks, the cost of testing, and follow-up.

### Finger Pricks

The finger prick is an integral part of the use of a BGM. Caregivers pointed out the problem that the children must prick themselves and underlined this with the fact that in children, this gesture could be easily associated with fear, screaming, and crying. They also mentioned the large number of pricks per day between blood glucose levels and insulin injections.

*Sometimes they do not want to prick their hands because they are hard, calloused and, as they are thin, they have no muscle mass, so it hurts. Imagine the trauma of pricking their fingers to measure glucose, to take insulin, imagine the life they lead*.

[P4, caregiver of a 12-year-old child]

Moreover, some caregivers even shared their self-fears of pricking their own child.

*The challenge comes for example when the patient condition worsens and for me I can’t prick her to read the test. I tell her sister to prick her because me I get scared,...*

[T1, caregiver of a 15-year-old adolescent]

However, the number of finger pricks for blood glucose measurement per day varied among interviewees, and consequently the burden this represented. In Kyrgyzstan, caregivers spoke of an average of 7 to 10 pricks per day, while in Mali, Tanzania, and Peru, it was more like 2 to 4 per day. M2 highlights the impact that 2 finger pricks a day can have on minors.

*He also refused to check his levels twice a day. He said it was being pierced all over his body. He was tired. He didn't want it anymore*.

[M2, caregiver of a 16-year-old adolescent]

Despite this, the BGM is appreciated for its simplicity and the ease with which it can be used by a child.

*She is just testing herself, whenever she feels like she is not alright she tests*.

[T4, caregiver of an 8-year-old child]

### Follow-Up

Blood glucose levels are a constant that varies throughout the day and night. One of the responsibilities of the caregivers is to be involved in this monitoring. However, more autonomous children prick themselves.

*Often when he gets a problem it is from not observing the test results. He makes haste to inject then eat. That’s where harm comes. So right now, I’m very careful, especially testing through the evening. I want when he measures, I see [the result]*.

[T6, caregiver of an unknown age child]

In addition, caregivers mentioned the necessity to accompany the child or teenager when blood glucose levels are measured at night, and K6 underlined the consequent sleep issues.

*Since my son got sick,...I go to bed late and wake up early. At night I have interrupted sleep....At night when his glucose rises and he sleeps I inject insulin, then I observe how insulin acts*.

[K6, caregiver of a 12-year-old child]

### Affordability

With regard to the cost of BGMs, some caregivers mentioned donations, for example, from health facilities, diabetes associations, or international programs such as Life for a Child or Changing Diabetes in Children, covering part of their needs.

*We didn't buy anything; they give us the devices and take care of everything. They give us the insulin, the injections and also the strips*.

[M2, caregiver of a 16-year-old adolescent]

Sometimes families still had to buy extras or make up for the limited amount of donated equipment.

*Yes, if you don't skip days just a few days they [strips] are over*.

[T4, caregiver of a 9-year-old child]

However, most interviewees mentioned that there are many costs involved in managing diabetes.

*Financial problems come first. Diabetes is not a disease when you have a normal financial situation*.

[K3, caregiver of an 8-year-old child]

Different means of saving money are discussed by the interviewees, including, for example, reusing lancets as described by P3.

*...we are not in a position right now to be spending lancets all the time because no, it is money, it is not possible*.

[P3, caregiver of a 12-year-old child]

### CGMs

The following results highlight the use of CGMs by the interviewees.

### Acceptability

The use of CGMs allows for fewer finger pricks, blood glucose monitoring at shorter intervals, monitoring from a distance and during the night, and better control of glycated hemoglobin, as underlined below by K2.

*Makes life easier because every time you monitor your child, running after the child to pierce her finger and check the glucose is a big problem for us. Before the sensor, the problems for us were to check her glucose at night. I had to wake her up, she would cry, and the mood would change for the worse. Now it’s much better with the sensor. Thanks to the sensor, our level of glycated glucose decreased from 11 to 9. This is because we quickly bring glucose to normal*.

[K2, caregiver of a 4-year-old child]

The reliability of the device is essential for caregivers. T3 said that he would adapt his use of the device according to his knowledge of his child’s condition.

*(Laughing) I would trust them [new devices] a bit...I will look also on the child’s condition since I have experience, I can now know that now it [blood glucose] has dropped and now it has risen. So, I will trust it depending on my experience of taking care of him*.

[T3, caregiver of an 18-year-old adolescent]

Caregivers underlined the problem that CGMs do not completely prevent finger pricks. P2 underlines the fact that some CGM devices require regular calibrations, and thus the use of finger pricks and the use of a glucose meter.

*Yes, at least twice a day because my daughter uses an insulin pump, and her pump, the sensor that her pump uses requires two calibrations a day, mandatory*.

[P2, caregiver of a 9-year-old child]

The length of use before changing the device is a crucial point for caregivers, who count their expenses monthly, and a 14-day device is recognized as being too short.

*...you know what depends there are two things, although my financial capacity is limited, but it also depends on how long the device can last?*

[T6, caregiver of an unknown aged child]

In addition, CGMs will have to be adapted to the cultural contexts and activities of the minors, as highlighted below by M6 and M5.

*Yes, [discretion] would be very important, because illness is something, even when people know you have such and such an illness, it will complicate your marriage*.

[M6, caregiver of a 15-year-old adolescent]

*Something that’s not a part of your body, God didn't create you like that, it’s not a shirt that you wear, except something that’s permanently stuck to your body and that marries with the blood, I think that can often bother sick people*.

[M5, caregiver of a 17-year-old adolescent]

### Accessibility

Most caregivers (except K6 and P4) owned a smartphone and therefore had potential access to the technology needed for CGM devices. A second condition for access was dependent on the caregivers’ knowledge of these new technologies. In Mali and Tanzania, none of the caregivers had heard of any device other than BGMs for measuring blood glucose levels. Another prerequisite is the skill set needed to use it correctly, requiring therapeutic education for caregivers, as expressed by P4.

*...it’s not just to say "look, here, I'll give you a glucose sensor and that’s it, put it on", I mean, first educate me, right? I mean, teach me, explain to me how it works, in other words, what use I can get out of the device, right, in other words, to make 100% use of it, not just, not just not pricking, not just putting it there to watch all day, in other words, what’s the point of seeing a horrible graph above 200, 300 and not knowing how to improve it*.

[P4, caregiver of an 11-year-old child]

There is also the problem of accessibility on the market and the availability of devices in pharmacies.

*Ah...Well, it would have to be coordinated between them, between the Ministry of Health and the people who manufacture the products, so that the products can be delivered, and people can buy them*.

[P6, caregiver of a 16-year-old adolescent]

### Affordability

CGM devices represent a significant cost for the families who use them. To save money, some people buy the devices abroad. K3 underlined competitive prices abroad.

*It would be nice if the sensor is sold here at the price that is sold in Russia. Now our company [name of company] sells a sensor for 6300 soms [approx. US$ 70]. In Russia, we buy [name of sensor] for 4300 rubles [approx. US$ 46]. You can order it for 3700 soms [approx. US$ 41] from France*.

[K3, caregiver of an 8-year-old child]

Others extend the use of devices as mentioned by K4.

*In general, you need to replace the sensor every 14 days, but our mothers have found a way to extend the life of this sensor, so of course we extend it. This saves our money*.

[K4, caregiver of a 9-year-old child]

However, K1 mentioned the savings they make using a CGM on other materials used for BGM.

*Before the sensor, we spent 4,000 soms [approx. US$ 45] for test strips. Now when we installed the sensor, we buy test strips for 2,000 soms [approx. US$ 22]*.

[K1, caregiver of a 12-year-old child]

Overall, for those who had never used CGMs, the main issues were to ensure that any new technology represented less than or the same expense as what they currently use. Realizing the significant costs involved, caregivers are prepared to pay. T4 spoke about the possibility of organizing themselves and involving family members to raise money to afford such devices.

*We will sit together and see how it’s sold…maybe and the siblings will contribute money for purchasing it*.

[T4, caregiver of a 9-year-old child]

A summary of the results related to BGMs and CGMs is presented in Table 4.

**Table 4.** Summary of findings.

Barrier/challenge	BGM^a^	CGM^b^
Price	High and total price is dependent on number of tests	Unaffordable for many; high upfront cost
Ease of use	Easy and familiar to use	Needs further knowledge and education to be used effectively
Psychological stress for caregivers	Familiarity with use; confidence in equipment	Need to become familiar with the equipment, education; ability to monitor blood glucose trends; alarm functions
Purchasing access	Available everywhere	Not available formally in the countries included in this study
Technology access	Smartphones not needed; electricity not needed as battery dependent	Technology dependent
Glycemic control	Measurement-dependent, highly reliable instantaneous value	Continuous readings, including nights, alarm systems, and remotely accessible data for caregivers
Finger prick “trauma”	Each time	At installation and for calibration
Aspects and activities of daily life	Provides a reliable measurement for adapting activities (nutrition, sports), requires dedicated time and handling, equipment to be carried with the individual, requires caregiver to be present (depending on the child's autonomy)	Allows continuous measurement of blood glucose levels in the event of variations (diet, sport), no special measuring effort or additional equipment required, remote control (practical for caregivers)
Self-education	Use of actual blood glucose values recorded in a notebook, no technology needed, requires many values	Better understanding of blood glucose trends, values may be incorrect and therefore require knowledge of hypoglycemia symptoms
Confidence and acceptability	High confidence and acceptability	Need for more information, good confidence among CGM users, issue of visibility of device

a BGM: blood glucose meter.

b CGM: continuous glucose monitor.

### Impact on Everyday Life

Beyond the actual tools, the management of type 1 diabetes has wide-ranging impacts on these families. SMBG is impacted by and impacts such aspects as daily life activities, nutrition, school, and access to medical care. Glycemic control involves adaptations to daily life for children, teenagers, and caregivers. K6 highlighted the impact of the condition on their lives.

*Diabetes is an incurable disease. Living with diabetes is to be 24 hours in tension. There are no rest and holidays with this illness. For me personally this disease was a huge stress. My life has fully changed, and my relationship with my son has become also different as he is now in his awkward age. I am alone and it is difficult for me morally and financially*.

[K6, caregiver of a 12-year-old child]

K4 emphasized the role of the caregiver and the stress experienced.

*She is a child, she does not understand, but for parents, this is a big burden and stress*.

[K4, caregiver of a 9-year-old child]

P3 and T6 also shared the need to supervise the reading of blood glucose results.

*...in fact, it was more of a challenge for me than anything else, it taught me that I have to be aware of my child, that I have to supervise him constantly...*

[P3, caregiver of a 12-year-old child]

Overall, the main issues impacting daily management of type 1 diabetes mentioned by caregivers were nutrition, school, and access to medical care.

### Nutrition

Caregivers encountered difficulties with imposed dietary restrictions linked to glycemic control and the child’s desires. K3 spoke about limiting food quantities and its impact on the child and the parent’s role.

*For mothers, it is psychological stress. It is also difficult to restrain the child in eating. She wants everything, and sometimes we can’t refuse, then hyperglycemia happens. Everything depends on us. You also need to learn how to calculate properly bread units*.

[K3, caregiver of an 8-year-old child]

Moreover, the diet involves efforts in terms of cooking, habits, and costs, as underlined below by K1, P3, and T4.

*Food restrictions is our problem. Another nuance is that expenses have increased, and for food too. Now we need to maintain proper nutrition, but it turns out to be much more expensive. The sugar substitute costs 5-6 times more than the sugar itself*.

[K1, caregiver of a 12-year-old child]

*And that’s not all because it also depends on the quality of the food because it’s not...it’s not a food, for example, of eating rice with eggs, you have to give it fibers, you have to give it vitamins*.

[P3, caregiver of a 12-year-old child]

*The problems I get are on diet. On diet some people...since I’m usually at work so that time of...because he has to eat four meals. So time for preparing him food becomes a problem and sometimes he doesn’t get those four meals. He can get three meals until I have gone extra miles to force him learn to cook on his own*.

[T3, caregiver of an 18-year-old adolescent]

Some parents mentioned the need to keep an eye on blood glucose results so as not to miss out on any dietary “misbehavior” on the part of the child.

*...for me whenever I doubt that there is a possibility that she might lie to me, I tell her after you finish to test then show me the reading from the machine*.

[T1, caregiver of a 15-year-old adolescent]

### School

During the day, SMBG of nonautonomous young children who go to school can be a real challenge for caregivers. K2 highlighted the adaptation of professional activities to be closer to the child.

*[The mother] specifically applied for [a] job in [a] kindergarten to keep track of [her] child*.

[K2, caregiver of a 4-year-old child]

K3 underlined the need for a child’s autonomy with a BGM.

*Since September this year, when she went to school, my daughter began to use a blood glucose meter on her own. It was time for her to learn because I can’t be with her all the time*.

[K3, caregiver of an 8-year-old child]

For some children, blood glucose levels are not monitored during the school day, as M4 mentioned.

*They're children, and I don't even allow her to bring the [BGM] to school because when they're playing with the other children, they might break it!*

[M4, caregiver of a 17-year-old adolescent]

Diabetes-related complications can arise at any time, and T4 mentioned the difficulties experienced with the child’s school.

*...They are scared because even now when she goes to school when she just feels bad, they tell me to go and take the child as she feels bad*.

[T4, caregiver of 9-year-old child]

Extracurricular activities such as sports, which are part of a child’s development, create another challenge for blood glucose monitoring.

*Before sport, blood sugar levels are good. But after sport, whoosh, he goes into hypoglycemia. I really don't know how to deal with that*.

[M1, caregiver of a 15-year-old adolescent]

Not all caregivers felt that diabetes management was particularly restrictive in terms of the respective activities of the child and teenager, who can still lead a normal life according to them.

*No, it hasn't made any difference to her work or school life, she never misses a day unless we have an appointment. Apart from that, there’s no change in her daily life*.

[M1, caregiver of a 15-year-old adolescent]

### Access to Medical Care

Managing diabetes involves regular medical consultations in specialist centers and access to emergency services. Caregivers point out the geographical and financial factors of transport involved. M2 noted receiving funds from hospitals for transportation, which is provided by Santé Diabète through support from Life for a Child. In addition, medical care involves additional costs that depend on health systems and differ among countries.

*The hospital only gives 1000 CFA [US$ 1.66] to the patient....Otherwise, you've seen the distance yourself. So, you have to take a lot of fuel to get there and back, every time there’s an appointment*.

[M2, caregiver of a 16-year-old adolescent]

Proximity to care facilities is critical; P3 shared that they relocated closer to the hospital following their child's hospitalization, while T5 noted selecting a school based on its distance from a medical center.

*I live in Talara, I was being treated at the Talara facility, but my child required hospitalization because, I mean, he was in a delicate condition. He suffered a decompensation, and they sent me to Sullana, so until now I have been in the support hospital in Sullana and, what is it called, and well, I am still here because I am afraid that my child will deteriorate again. I am still here in Sullana*.

[P3, caregiver of a 12-year-old child]

*I sent him to these regular schools because outside the school there is Twangoma hospital*.

[T5, caregiver of a 13-year-old adolescent]

## Discussion

### Principal Findings

This study reports on the perceptions of caregivers on the use of BGMs and CGMs and the impact of SMBG. The concerns mentioned about blood glucose monitoring using BGMs and CGMs in children and adolescents living with type 1 diabetes included the issue of BGMs requiring repeated finger pricks and, for CGMs, the financial burden given their high cost. Device use differed substantially across the 4 countries, reflecting variations in economic factors of the countries and individuals. All participants in Kyrgyzstan used CGMs, whereas none of the caregivers in Mali or Tanzania reported access to CGM technology. CGMs remain more expensive than BGMs, but BGMs are also financially difficult to access without the donations that were available to people in Mali and Tanzania.

In addition to these device-related challenges, caregivers also mentioned the psychological distress related to the management of diabetes, concerns about their child’s health and blood glucose control, and the medical and medication costs they incurred. SMBG was reported as having wide impacts on daily elements such as diet and children monitoring blood glucose at school. The factors that appeared to alleviate the burden on caregivers were familiarity with type 1 diabetes, including acquired knowledge and recognition of hypoglycemia symptoms, the child’s increased autonomy, and support from family. For those children using CGMs, the interpretation of their caregivers’ perspective was that CGMs also served to lessen the burden of type 1 diabetes management.

In terms of the child’s autonomy, CGMs were reported by interviewees to play a valuable role in supporting this aspect. Respondents stated that CGMs helped the child better understand their hypoglycemia symptoms, continue extracurricular activities, reduce the need for frequent finger pricks, and more effectively manage their food intake and insulin dosage. CGM users appeared to be highly satisfied with the support these devices provided in their daily lives. Interviewees particularly highlighted the reduction in finger pricks, continuous monitoring (including overnight), valuable assistance in the management of type 1 diabetes, and the resulting alleviation of the psychological burden.

### Implications

Differences in access to self-monitoring across countries likely reflect broader structural inequities in procurement systems, insurance coverage, and supply chains, as previously documented in LMIC health system assessments [12]. These disparities suggest that technological innovation alone will not reduce caregiver burden without parallel financing and policy reform. To implement CGMs in the management of type 1 diabetes in children and adolescents, the focus must be on their accessibility (market availability, medical knowledge, and therapeutic education), their affordability, as well as their acceptability, feasibility, and sustainability.

Beyond the role technology can play for the child or adolescent, its impact on caregivers should not be neglected, given that parents play a key role in managing their child’s condition on a daily basis [27]. Blood glucose levels in diabetes are impacted by multidimensional factors and are particularly demanding, requiring major adaptations to daily life. The effective management of these different measures in children and adolescents is a major challenge for the parents concerned, as they have control over many of these elements, such as food, supplies, medication, appointments, sports, school enrollment, and transportation. Existing literature in South Africa has also shown difficulties in terms of caring for children with diabetes, such as emotional, social, practical, and financial difficulties [28]. Raina et al [29] have shown that higher caregiving demands were associated with lower psychological and physical health among caregivers of children with chronic conditions. Several parent-related determinants, such as poor mental health, uncertainty about illness, and poor self-efficacy, can negatively impact their ability to monitor their child’s glycemic levels effectively, ultimately leading to suboptimal diabetes management in children [30,31]. A qualitative study conducted in China highlighted some of the challenges faced by caregivers of children newly diagnosed with type 1 diabetes, such as psychological stress, difficult family functioning changes, daily management, financial burden, and the lack of social support for parental caregivers [32].

CGMs have been touted as a means to support better blood glucose monitoring [6,7]. A study on the role of CGMs carried out in Pakistan among a population of people living with diabetes and mostly (90%) using BGMs found that a majority believe in the role that technology can play in supporting management of diabetes by improving care, reducing complications, and simplifying monitoring [33]. The study also highlighted the affordability problem, which seems to be a major barrier due to high out-of-pocket health-related expenses. Burckhardt et al [34] were able to show that the use of remote monitoring with CGMs significantly improved the daily experience of caregivers and increased their feeling of a better understanding of diabetes care and control. CGMs are among the emerging technologies designed to improve diabetes management, particularly by enabling better glycemic control and reducing glucose variability. They are now widely used in high-income countries and have demonstrated positive outcomes, especially among children and adolescents, including improved time-in-range and reduced hypoglycemia [35]. These elements, as well as the results from this study, can inform target product profiles and how tools for SMBG can be adapted to different settings, incorporating not only expert views [36] but also those of people living with diabetes and their caregivers. Clearly, in LMICs, cost is an element that needs to be addressed. Other factors include the visibility of the sensor, operation across different ambient temperatures, alert functions, and overall ease of use [21]. Beyond these technological factors, this study highlights how technology needs to be integrated with the other factors that caregivers and people with type 1 diabetes must manage on a daily basis.

### Limitations

This qualitative study has several limitations in terms of data collection, sample size, and sampling, as the interviews were conducted by different people in different countries, and the people interviewed were selected by the respective organizations in each country. Also, in many contexts, the issue of how representative of all people with diabetes the sample was should be considered, in terms of exposure to diabetes-related technologies, knowledge of diabetes, as well as socioeconomic factors. Moreover, there may have been biases in analysis and results presentation linked to the researchers’ different knowledge and perspectives on the topic. Finally, the analysis was conducted by a researcher with little knowledge of the local cultures in 3 of the 4 countries, as well as limited lived experience of type 1 diabetes. This may have resulted in contextual bias in analyzing and presenting the data.

### Conclusions

This study contributes to the limited qualitative evidence on the perceptions of caregivers in LMICs with regard to blood glucose monitoring and the impact of the tools used to manage it. Blood glucose monitoring is a cornerstone of diabetes management to prevent morbidity and mortality. As highlighted in this paper, many barriers exist with regard to both the daily burden of SMBG, as well as the cultural, technological, and cost-related aspects of the tools needed to do so. With the first-ever global diabetes coverage targets, including “80% of people with diagnosed diabetes have good control of glycemia” and “100% of people with type 1 diabetes have access to affordable insulin and blood glucose self-monitoring” [37], addressing all these barriers to proper SMBG is necessary.

SMBG is a cornerstone of type 1 diabetes management. For children and adolescents, caregivers play an important role in all elements of diabetes care, including blood glucose monitoring. Thus, their perceptions are important to ensure that the tools offered also respond to their needs and to those they perceive for their children. Device characteristics need to meet requirements for reliability, durability, and context-specific acceptability to children, adolescents, and their families. Beyond being adapted to the needs and context, a clear barrier to the use of BGMs and especially CGMs is their cost. Whereas CGMs are a given in high-income countries, in LMICs they remain accessible only to those with the means to pay for them, in many cases abroad, as they are not registered or readily sold, as, for example, in Kyrgyzstan. These tools also require patient education and empowerment through trained health professionals to ensure that the cost of self-monitoring tools has a positive impact on blood glucose control, as well as on the mental health and well-being of the person with type 1 diabetes and their caregivers.

## Abbreviations

ACCISS: Addressing the Challenge and Constraints of Insulin Sources and Supply
BGM: blood glucose meter
CGM: continuous glucose monitor
LMIC: low- and middle-income country
SMBG: self-monitoring of blood glucose
